# Seed Cryopreservation, Germination, and Micropropagation of Eastern Turkeybeard, *Xerophyllum asphodeloides* (L.) Nutt.: A Threatened Species from the Southeastern United States

**DOI:** 10.3390/plants10071462

**Published:** 2021-07-16

**Authors:** Michelle Issac, Princy Kuriakose, Stacie Leung, Alex B. Costa, Shannon Johnson, Kylie Bucalo, Jonathan M. Stober, Ron O. Determann, Will L. Rogers, Jenifer M. Cruse-Sanders, Gerald S. Pullman

**Affiliations:** 1 School of Biology, Georgia Institute of Technology, Atlanta, GA 30332, USA; mmissac@gmail.com (M.I.); princyk730@gmail.com (P.K.); Stacie.leung@gmail.com (S.L.); alexbcosta87@gmail.com (A.B.C.); 2 Renewable Bioproducts Institute, Georgia Institute of Technology (Formerly the Institute of Paper Science and Technology), 500 10th Street NW, Atlanta, GA 30332, USA; smjohnson777@yahoo.com (S.J.); kbucalo@ohanainstitute.org (K.B.); 3 US Forest Service, USDA, Talladega National Forest, Shoal Creek Ranger Dist, Heflin, AL 36264, USA; jonathan.stober@usda.gov; 4 Department of Conservation Research, Atlanta Botanical Garden, 1345 Piedmont Ave., NE, Atlanta, GA 30309, USA; rondetermann@gmail.com (R.O.D.); crusesanders@uga.edu (J.M.C.-S.); 5 State Botanical Garden of Georgia, 2450 Milledge Avenue, Athens, GA 30605, USA; bonjour@uga.edu

**Keywords:** conservation, cryopreservation, endangered species, micropropagation, shoot culture, *Xerophyllum asphodeloides*, Eastern Turkey Beard

## Abstract

*Xerophyllum asphodeloides* (Xerophyllaceae), known as eastern turkeybeard, is an herbaceous perennial found in eastern North America. Due to decline and destruction of its habitat, several states rank *X. asphodeloides* as “Imperiled” to “Critically Imperiled”. Protocols for seed cryopreservation, in vitro germination, sustainable shoot micropropagation, shoot establishment in soil, and seed germination are presented. Seeds from two tested sources were viable after 20 months of cryopreservation. Germination of isolated embryos in vitro was necessary to overcome strong seed dormancy. Shoot multiplication and elongation occurred on ½ MS medium without PGRs. Shoots rooted in vitro without PGRs or with 0.5 mg/L NAA or after NAA rooting powder treatment and placement in potting mix. When planted in wet, peaty soil mixes, shoots grew for two months and then declined. When planted in a drier planting mix containing aged bark, most plants continued growth. In the field, plant survival was 73% after three growing seasons. Safeguarding this species both ex situ and in situ is possible and offers a successful approach to conservation. Whole seeds germinated after double dormancy was overcome by incubation under warm moist conditions for 12 weeks followed by 12 weeks cold at 4 °C and then warm.

## 1. Introduction

*Xerophyllum asphodeloides* (L.) Nutt. is a monocotyledonous herbaceous perennial species in the Xerophyllaceae, Beargrass Family, ref. [1] within the Liliales [2]. One of two species in the genus, *X. asphodeloides*, also known as eastern turkeybeard, is native to eastern North America where it is found in the Pine Barrens of New Jersey and also in the Appalachian Mountains of Georgia, Carolinas, Virginia, West Virginia, Tennessee, and Alabama and is presumed extirpated in Kentucky and Delaware [3,4]. The two habitats share features of dry, acidic, nutrient poor, and sandy or gravelly soils.

Threats for this species include trampling by hikers, collection as a homeopathic remedy, conversion of Pine Barrens to residential properties, and fire suppression leading to succession of wood canopy [4]. Loss of habitat has threatened the presence of this species and increased its risk of endangerment. *X. asphodeloides* is currently ranked as G4, being “Apparently Secure”. However, due to the decline and destruction of its habitat, several states, including West Virginia, Alabama, and Georgia, rank *X. asphodeloides* as S1 (Critically Imperiled) and South Carolina rank it as S2 (imperiled) [4]. In 2001, the USDA reported that *X. asphodeloides* could be found in a total of 41 counties across its range of habitat [4]. In Georgia, 10 populations are known, with 2 on national forest land and 3 on state conservation lands [5]. In Alabama, 16 populations are known to exist in one county [4].

Conservation of this species is important due to its ecological relationships. As to its co-existence with many other plant species, threat to *X. asphodeloides* may have a parallel effect on its habitat. Additionally, it has been significant for medicinal use in homeopathic remedies treating constipation, dysmenorrhea, and skin irritations [6]. Research on its congener, *X. tecta*, indicates that culturally relevant management of the landscape through indigenous fire stewardship and leaf harvest for weaving positively impacts demographic processes and changes in long term practices result in population declines [7,8]. Cryopreservation has been used for seed and shoot tip conservation, providing long-term storage [9,10,11,12], and is considered to be the best long-term approach to preserving endangered species [13].

In vitro techniques can be used to multiply rare or endangered plants asexually [12,14,15,16]. Micropropagation has many advantages, including rapid production of identical plants. Under in vitro conditions, many disease-free plants can be produced in a small space that are often more robust and show accelerated growth compared to similar plants produced by seeds or cuttings. With careful planning to manage genetic source material, plants of endangered species produced through micropropagation can be used to preserve genetic diversity, supplement small populations, or to establish new planting sites for conservation, research, education, or recreational purposes.

A simple method for whole seed germination is required for plant production from cryopreserved seeds. At the time this research began, information on *X. asphodeloides* dormancy was not available. Reports indicated that seeds cannot tolerate long term desiccation and seed germination in the greenhouse and field only occurs after two growing seasons (19,20). In 2014, based on the report of Brumback (19), Baskin and Baskin (29) classified *X. asphodeloides* as having morphophysiological dormancy where the seed contains an undeveloped embryo and germination is initiated after 4 weeks of incubation.

The objectives of this research were to develop reliable protocols for cryogenic storage of seeds and micropropagation to preserve this species for the future using seed to start cultures. A good protocol for germination was required to recover plants from cryopreserved seeds. Through a series of experiments, three approaches to ex situ conservation were tested: (1) in vitro germination of seeds and seed cryopreservation for long term storage, (2) germination of isolated embryos in vitro to produce plants for micropropagation, and (3) results from in vitro experiments led to testing methods to form rooted shoots and establish them in soil.

## 2. Results

### 2.1. X. asphodeloides Seed Sterilization and Germination Experiments

Germination did not occur in Section 4.2.1, Section 4.2.3, Section 4.2.4, Section 4.2.5, Section 4.2.6, Section 4.2.7, Section 4.2.8, Section 4.2.9 and Section 4.2.10. In the more than 1000 seeds that were tested for germination the only treatment that contained any germinants was with the application of 4 min sulfuric acid as a scarification treatment in Section 4.2.2. Most tests with H_2_O_2_ surface sterilization or H_2_SO_4_ scarification showed little to no contamination. Scarification is known to remove dormancy and also to decontaminate the seed surface [17,18]. Tests to repeat and optimize the scarification treatment in Section 4.2.5, Section 4.2.6, Section 4.2.7, Section 4.2.9 and Section 4.2.10 with source 2 seeds showed no germination.

Section 4.2.2. Seed scarification. In the first experiment, seeds scarified for 1 min with sulfuric acid showed the least seed coat damage, while treatment for 10 min caused almost complete removal of the coat. Seeds treated for 4 min showed some damage, and 90% of seeds scarified for 1 min with acid showed fungal contamination compared to no contamination when scarified for 4 or 10 min.

**Figure 1:**
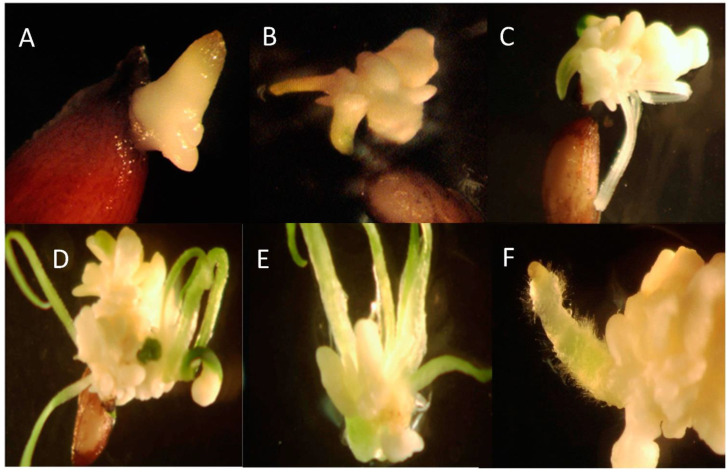
Seed source 1 *Xerophyllum asphodeloides* germinant from a 4-min H_2_SO_4_ scarification treatment. Days after seed began to germinate. (**A**) Day 1, (**B**) Day 26, (**C**) Day 36, (**D**) Day 92, (**E**) Shoot clump 3 weeks after division, (**F**) Emergence of a root from the growing tissue.

After approximately 30 days, two seeds in the 4 min treatment began to show emergence of an off-white tissue presumed to be the radical (Figure 1A). Two more seeds germinated later, for a total of 4 out of ten. Germinants appeared to form a cluster of tiny bulbs that continued to grow producing a bulbous tissue that after another month began to produce several shoots with strap-like leaves (Figure 1B–D) and roots (Figure 1F).

In the second experiment, the percentage of seeds with fungal contamination was high for short scarification times for source 1: 3 min (45%), 3.5 min (67%), 4 min (27%). Minor contamination was observed for source 2: 2 min (17%), 3 min (0%), 4 min (0%), 5 min (0%). However, no germination occurred, and seeds were scarified for 4 min and planted in greenhouse soil did not germinate.

### 2.2. Tetrazolium Chloride Seed Viability Staining

In the initial experiments, little or no germination occurred. We became concerned that seeds may not be viable. Seeds from sources 1 and 2 stained red after soaking in tetrazolium chloride for 48 h indicating seeds were viable. However, seeds that underwent a 10 min scarification did not stain.

### 2.3. X. asphodeloides Embryo Isolation and In Vitro Germination Experiments

With little success in germinating *X. asphodeloides* seeds, sporadic germination occurring after scarification and confirmation of viability, we hypothesized that seed coat-imposed dormancy prevented germination, and removal of the embryo from the seed would allow germination to occur.

**Figure 2:**
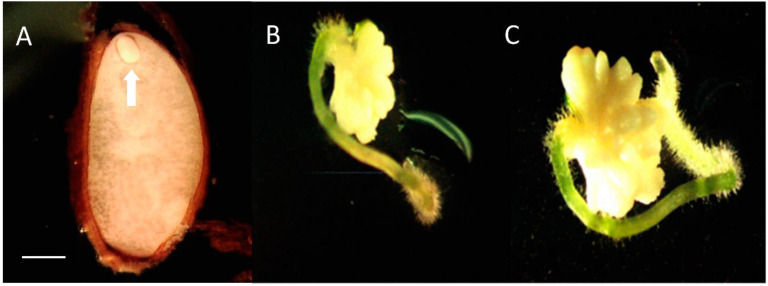
*Xerophyllum asphodeloides* seed with embryo. (**A**) Unstained embryo before isolation (arrow). (**B**) Embryo excised from sterilized seeds growing on ½ MS without PGRs medium 35 days after placement on medium. (**C**) 56 days after placement on medium. Scale bar = 1 mm.

Seeds contained small oval embryos that were isolated with forceps (Figure 2A). Percent contamination of the embryo cultures grown on ½ MS without PGRs, full MS without PGRs, ½ MS + NAA and full MS + NAA media was recorded: 23%, 14%, 24%, and 18%, respectively. After two weeks growth of the isolated embryo was visible. Embryos on ½ and full-strength MS medium without PGRs developed small roots and formed bulb-like structures that enlarged in size (Figure 2B,C). Embryos placed on media with NAA formed bulb-like tissue with or without pointy green structures. After four weeks presence of roots and/or bulbous tissue were 25%, 20%, 19%, and 3% for ½ M without PGRs S, full M without PGRs S, ½ MS + NAA, and full MS + NAA media, respectively. The best response with the most leaves and the least callus occurred on ½ MS medium without NAA.

### 2.4. Embryo Growth on ½ MS Medium with Different Sucrose Concentrations

Embryo germination for sources 2 and 3 increased as sucrose concentration increased with the highest germination percentage at 6%, however, differences were not statistically significant (Table 1). Source 1 embryo germination was similar for 3 and 6% sucrose (Table 1). Embryo germination for the five Alabama sites tested with 3 and 6% sucrose concentrations did not differ significantly, although the interaction between germination and seed source was statistically significant (Table 2).

### 2.5. X. asphodeloides Shoot Multiplication Experiments

**Figure 3:**
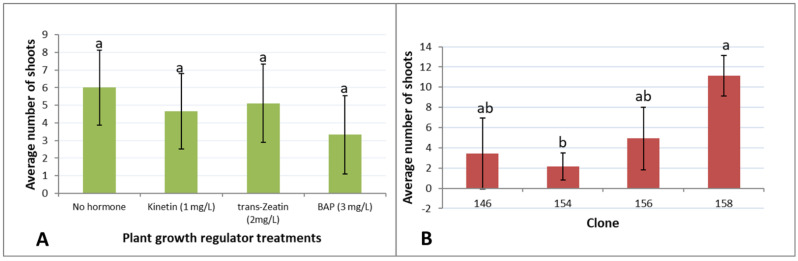
*Xerophyllum asphodeloides* shoot multiplication on ½ MS medium without PGRs after five weeks. Standard errors are shown for each treatment. Treatments with same letter are not statistically different by multiple range test at *p* = 0.05. (**A**) Effect of cytokinins averaged for the four clones. (**B**) Effect of clone averaged for the four media tested.

Shoots obtained from the four germinants in trial 2 grown on ½ MS without PGRs continued to grow and produce new shoots on all media tested, producing four clones each with multiple shoots. A few of the smallest shoot clumps did not survive. The highest number of shoots grew on ½ MS medium without PGRs, although differences were not statistically significant (Figure 3).

Thidiazuron had a strong effect on shoot multiplication over a four-month subculture. Low concentrations of thidiazuron (0.1 mg/L) influenced the shoot formation showing the highest number of shoots (Table 3). Differences were statistically significant (*p* = 0.05) with respect to control 1. DMSO, used as control n.2, did not show a statistically significant effect on shoot multiplication.

Thidiazuron again had a strong effect on shoot multiplication over a two-month subculture, in particular on shoots with minor size (Table 4). Shoot multiplication increased as thidiazuron increased with an optimal concentration at 0.15 mg/L and declined as thidiazuron was further increased. Differences were statistically significant for both small and large shoots (*p* = 0.05).

Shoot cultures grown on 0.15 mg/L thidiazuron for two 2–4-month subcultures continued to multiply but showed expanded and deformed shoot bases, suggesting that either thidiazuron concentration was too high or continuous exposure to thidiazuron is detrimental to shoot quality. On the contrary, shoots from all seed sources grown on PGR-free multiplication medium continued to produce new shoots during micropropagation cycles of 2–4 months over several years, suggesting shoot production is sustainable over time. With these results, further shoot multiplication was carried out without PGRs.

### 2.6. X. asphodeloides Root Induction and Acclimation to Soil

Shoots in the preliminary rooting experiment grew for about 3 months and then damped off and died.

**Figure 4:**
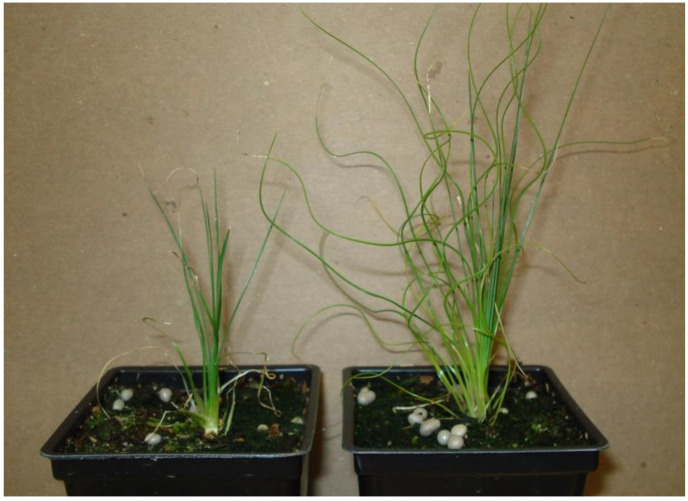
Shoots from isolated embryos from seed source 5 germinated in vitro and transferred to greenhouse soil under domes. Until two months plants grew well, but after 3 months they damped off and died.

**Figure 5:**
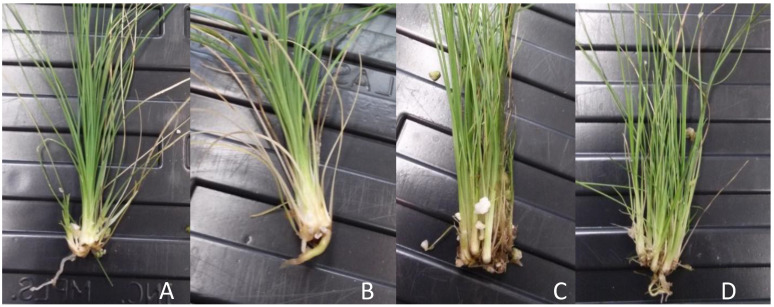
*Xerophyllum asphodeloides* shoot growth and rooting after 1 month in greenhouse potting mix. (**A**) DF-1 non-rooted. (**B**) DF-1 rooted. (**C**) LC-55 non-rooted. (**D**) LC-55 rooted.

**Figure 6:**
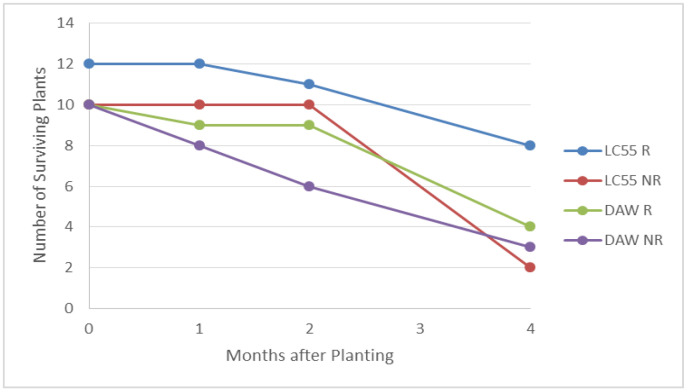
*Xerophyllum asphodeloides* micropropagated shoot survival over time for two clones planted in greenhouse soil. R (shoots with roots at the time of planting), NR (without roots when planted).

In the first experiment, after one month almost all shoots were still alive regardless of presence of roots at the time of planting (Table 5). Source 3 plants with roots at the time of planting appeared to be a bit stronger. Some non-rooted shoots from source 2 showed yellowing and some dead leaves but were still alive. Examined shoots in all treatments showed new root growth (Figure 4). After two months, many of the plants were showing new shoot and root growth (Figure 5). Between two and three months, shoots began to decline. At four months many of the plants had died. Shoots were considered dead if they were completely brown and withered. Almost all of the surviving plants had roots and were larger than when planted, but most were yellowing and showing dead leaves, suggesting continuing decline (Figure 6).

#### In Vitro Root Induction

With repeated observations of plant decline and death after two to three months in the greenhouse, we speculated that even though 80–100% of the plants formed roots, the sparse number of roots formed spontaneously or due to rooting powder treatment may be improved in number and vigor through in vitro rooting treatments with auxin.

**Figure 7:**
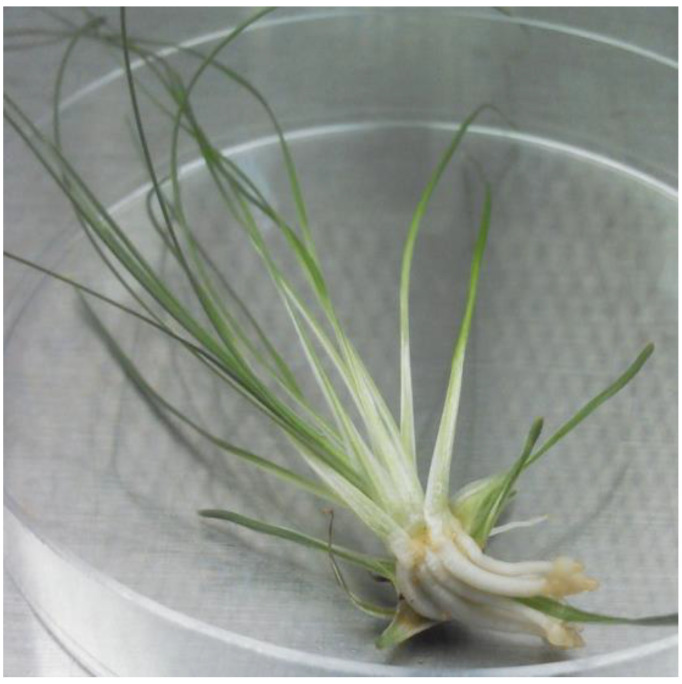
*Xerophyllum asphodeloides* after six weeks in 0.5 mg/L NAA rooting medium. Multiple vigorous roots have formed.

**Figure 8:**
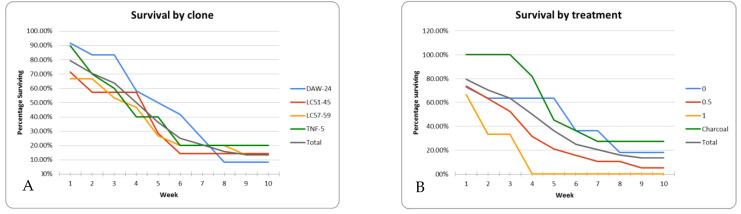
*Xerophyllum asphodeloides* micropropagated shoot survival over time after in vitro rooting treatments and shoots from four clones planted in greenhouse soil. (**A**) Survival by clone. (**B**) Survival by in vitro rooting treatment.

Roots in the in vitro rooting experiment began to develop at three weeks and over time root length, number of roots per shoot, and the percentage of shoots forming roots all increased (Figure 7). Maximum rooting percentages (90%) across the four clones (DAW, LC-51, LC-57, and TNF) occurred at nine weeks in the treatment containing 0.5 mg/L NAA (Table 6); differences between treatments were statistically significant at *p* < 0.01. Shoot rooting and health were evaluated prior to planting (Table 6). After nine weeks of in vitro rooting, the largest number of dead shoots occurred in the 1 mg/L NAA treatment suggesting that long term exposure to high NAA concentration reduced shoot health. Clone LC-51 also showed low survival across all treatments (60% died), likely due to presence of bacterial contamination. Shoots were planted in potting mix after nine weeks of in vitro rooting treatment. After planting, shoot survival steadily declined regardless of treatment (Figure 8).

With three prior observations of plant decline and death after two to three months in the greenhouse, we further speculated that once roots form they may require a better drained planting mix to better match the dry environment they grow in naturally to avoid damping off. *X. asphodeloides* normally grows in dry acidic, rocky, loose, well-drained soil with a northwest exposure [4].

The plants placed in SBG1 soil did very well, they survived and continuously grew over nine months. Plants did not exhibit the decline we usually saw after two months in peaty soils that were watered daily. This suggested that the drier environment and possible beneficial microorganisms contained in the aged bark improved growth and survival.

The native soil was very dense and would adhere to roots easily. ABG mix is rocky due to perlite and does not cling to roots like the native soil. SBG mix is more like a traditional soil, as it contains aged pine bark and adheres easily to the roots. All rooted shoots survived and continued to grow. Plant decline was not seen as in prior experiments with ABG potting mixes and daily watering. The reduced watering appeared to be beneficial. Addition of native soil did not improve plant growth or appearance. Plants in SBG1 soil mix exhibited faster growth with a greater number of leaves.

### 2.7. X. asphodeloides Acclimated Greenhouse Plants Planted in the Field

**Figure 9:**
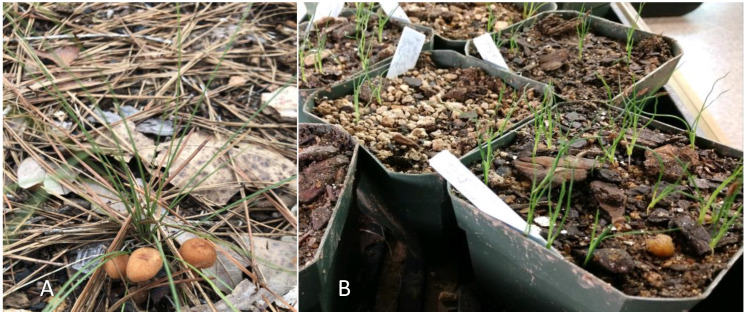
(**A**) *Xerophyllum asphodeloides* plants after one year in the field. (**B**) Whole seed germinated after prolonged stratification.

Plants transferred to the field survived and grew normally (Figure 9A). After 21 months of in-field growth, survival per plot was as follows: Plot A = 100%, Plot B = 57%, Plot C = 67%, Plot D = 63%, with an overall 73% survival. After two growing seasons plant size and leaf count were similar across all plots, with plants averaging about 20 cm in height.

### 2.8. X. asphodeloides Seed Cryopreservation

Water content of source 3 seeds was determined to be 10.2%.

Whole cryopreserved seeds did not germinate after LN treatment likely due to seed coat-imposed dormancy. For this reason, embryos were excised from cryopreserved seeds.

Twenty percent of embryos (20%) grew from cryopreserved seed, while none of the control isolated embryos grew.

Water content of seed sources 4 and 5 measured 5.63% and 4.61%, respectively. Cryopreserved and control seeds showed similar percentages of isolated embryo germination with averages for the two seed sources of 57% for control seed and 57% for cryopreserved seed (Table 7).

The additional seeds from sources 2, 3, 4, and 5 were evaluated for survival after varying periods of time at room temperature or after cryopreservation (Table 8). Seed sources varied, but seeds appeared to decline in viability with storage longer than 1 year at room temperature. Cryopreservation of viable seeds appeared to halt the decline as seeds from two sources cryostored for nearly two years after 4–5 months at room temperature showed survival similar to fresh non-cryopreserved seeds.

### 2.9. X. asphodeloides In Vitro and In Vivo Germination with Treatment for Double Dormancy

None of the seeds in medium or soil germinated after 12 weeks at room temperature or after the next period of 12 weeks at 4–5 °C. Seeds began to germinate only after the moist/warm and cold exposures ended and seeds were returned to room temperature. After 8 weeks in the final phase of room temperature, germination was recorded for the in vitro and in vivo treatments. Based on total seeds tested, both treatments showed similar germination percentages (Table 9). Seed source 2017-3 showed 50 and 47% germination for seeds germinated in medium vs. soil. Seed source 2017-5 showed 60 and 53% germination for seeds in medium vs. soil. Microbial contamination in medium was high for both sources with 53 and 40% for sources 2017-3 and 2017-5, respectively. Overall germination percentages in medium or soil did not differ significantly. Since seeds were surface sterilized, it is interesting to note that almost all seeds germinated on medium when not contaminated. Contaminated seeds in soil likely did not germinate and therefore overall germination percentages were similar in both treatments due to the loss of seed viability from microbial contaminants within the seeds.

When seeds in moist sphagnum moss were removed from the cold after 16 weeks, surprisingly, three yellow-leafed germinants were present. After planting in SBG1 mix and placement in the lighted greenhouse, the yellow germinants turned green and continued growing.

Seeds placed in bags of sphagnum moss at 4–5 °C in the dark began to germinate after 14–16 weeks of stratification. When moved to grow lights and later planted in 10 cm pots germination continued to occur, with final germination shown in Table 10 and Figure 8B. Note that seeds were stored dry for eight months at room temperature and had probably lost some viability from storage. Germination percent ranged from 0 to 23 and nine of the 11 seed sources showed some germination confirming our prior observations of germination after prolonged stratification.

## 3. Discussion

*Xerophyllum asphodeloides* is an interesting, important, and little-studied species that is threatened by fire suppression and the loss and fragmentation of its habitat. We report here, for the first time, cryopreservation and micropropagation procedures for *X. asphodeloides.* There is little information on the germination of *X. asphodeloides*. Some treatments are shown to overcome coat-imposed seed dormancy both in vitro or in vivo, using an embryo isolation method or whole seeds.

Brumback [19] categorized *X. asphodeloides* as a species that must be sown immediately outdoors upon collection and may take two years to germinate if freshly sown. Cullina [20] classified *X. asphodeloides* as a semi recalcitrant or hydrophilic seed type that does not tolerate dry storage and must be sown immediately upon ripening. A category of seeds intermediate between orthodox and recalcitrant is recognized in which seeds survive desiccation, but are damaged during dry storage at low temperatures [21].

Our research shows that *X. asphodeloides* seeds lose viability over 1–2 years of dry storage. Cullina [20] suggested the seeds are hydrophilic and do not take desiccation very well. Desiccated seeds stored at room temperature may lose viability while seeds kept moist in plastic with cool temperatures or in a soil bank may remain viable for years [22]. This condition is a problem for seed conservation [18,23,24,25]. Cryopreservation of seed, somatic embryos, plant meristems, or other tissues is an excellent tool for germplasm safekeeping [10,11,12,26,27]. The first set of cryopreserved seed did not germinate likely due to seed coat-imposed dormancy. The 2nd set of seed was 13 months old and probably lost viability over time. However, 20% survival in the 2nd set of seeds after cryopreservation indicated that *X. asphodeloides* seeds can tolerate cryopreservation. The 3rd cryopreservation experiment with fresher and drier seeds (stored 3–5 months at room temperature with 4.6–5.6% water content) showed similar embryo germination before and after cryopreservation as well as cryostorage prevention of viability loss over 20 months. Optimum moisture content for seed cryopreservation varies from 7 to 14% depending on species and seed lipid content [9]. Our determined seed water contents of 4.6–10.2% allowed successful cryopreservation of *X. asphodeloides* seeds.

Seed dormancy will often prevent germination even if the environment is fine. Seed dormancy provides additional time for a seed to disperse over geographic distances. Two types of dormancy occur: embryo dormancy often due to the ratio of gibberellins to abscisic acid (ABA) and dormancy imposed by the seed coat, endosperm, or pericarp [28]. For the latter, embryos will usually germinate once the seed coat and surrounding tissues have been damaged or removed. To overcome seed-coat dormancy and germinate, seeds often require long-term cold exposure while moist or damage to the outer tissues by long-term microbial activity, mechanical abrasion or contact with strong acids in the digestive system of an animal [29]. Acid or mechanical scarification is often used to speed germination of many seeds with thick coats. The ABA/gibberellin (GA) balance theory suggests dormancy in some species is controlled by antagonistic effects between ABA and GAs. Long-term cold stratification has been shown in some species to increase GAs that counteract germination inhibitors such as ABA [30]. Conditions that occur during moist stratification at 4 °C or chemical scarification can thus break dormancy by altering the ABA: GA ratio and/or thinning the seed coat to increase chemical diffusion and decrease coat strength.

Usually, germination in vitro is simple and involves surface sterilizing seeds in bleach or hydrogen peroxide and the seed germinates on medium within a few weeks. With *X. asphodeloides* this has been a difficult step because we did not have information about its germination requirements. Several methods to stimulate germination were tested including: MS medium salt strength, scarification, cold exposure, GA and or fluridone exposure, ABA exposure, heating exposure, smoke water exposure, and long times on germination medium. Out of about 1000 seeds, only a few germinated and these occurred when seeds were acid scarified suggesting seed coat-imposed dormancy prevented germination. However, further testing will be necessary with seed acid scarification alone or combined with other dormancy-breaking treatments to obtain high seed germination percentage

After numerous preliminary tests to germinate intact *X. asphodeloides* seeds in vitro, we decided to apply a different approach sometimes used to overcome interspecific incompatibility for rare lily hybrids [31]. *X. asphodeloides* seeds were surface-sterilized followed by dissection and removal of the embryo. Because dormancy is often caused by the seed coat and tissues surrounding the embryo, ref. [29] isolation of the embryo followed by placement in vitro on medium can sometimes remove dormancy. This approach worked well for *X. asphodeloides* and hundreds of embryos germinated in the trials carried out on ½ MS medium without plant growth regulators.

Seedling or shoot tip culture in vitro is commonly used to propagate plants for agricultural, conservation, horticultural, or medicinal purposes. In particular, cells from shoot tips are generally genetically stable and shoot tip culture can produce identical copies of target plants. Propagation with other tissue culture systems involving callus, organogenesis or somatic embryogenesis may induce somaclonal variation [15,32]. Since our goal is to maximize natural variation and minimize in vitro-induced variation, seed and shoot tip culture methods can provide excellent methods for preservation and reintroduction of *X. asphodeloides*.

*X. asphodeloides* shoots from germinated seeds and isolated embryos were tested for multiplication with increases of about 6 new shoots on ½ MS PGR-free medium or 15 shoots on medium with 0.1 mg/L thidiazuron over 2-month subculture cycles. Tests over 12 months showed sustainable shoot multiplication on PGR-free medium. Thidiazuron is a diphenyl-substituted urea and is often used for woody plant tissue culture due to its potent cytokinin activity [33]. Low concentrations of thidiazuron can stimulate axillary shoot proliferation while higher concentrations can stimulate axillary and adventitious shoot formation. Thidiazuron has also shown promise for micropropagation of some recalcitrant species in the Liliaceae and other plant families [34,35].

Rooted shoots from in vitro shoot cultures began to grow easily under domes in a greenhouse when watered daily or sparsely every other day. However, plants in soil mixes watered daily grew for several months and then declined, while light watering supported high survival and continued plant growth. We speculate that daily watering causes damping off and root rot while light watering allows the surface soil to dry out creating a more natural environment for root growth. In addition, the use of substrate, the aged pine bark in SBG 1, may harbor beneficial microorganisms that help *X. asphodeloides* roots to thrive. Abundant research with micropropagated plants supports the use of bio-agents to improve shoot acclimatization, survival and growth [36,37,38].

Seeds from some species have very specific germination requirements. For example, some genera in the Liliaceae and Melanthiaceae have species with deep simple double morphophysiological dormancy [29]. Baskin and Baskin [29] reported that seeds of *X. asphodeloides* take two winters to germinate during which the embryo grows to a critical size followed by exposure to environmental conditions that break physical dormancy [29].

When *X. asphodeloides* seeds germinated in a greenhouse, germination usually took more than a year before a shoot emerged from the soil [22,39]. Our findings along with observations of Brumback [19] and Cullina [20] suggest that *X. asphodeloides* is an intermediate type seed with morphophysiological dormancy (MPD) and dormancy can be overcome by exposure to warm moist conditions followed by cold stratification followed by continued cold or warm moist again. While nine levels of MPD are recognized (29), more research is needed before we clearly understand the type of MDP present in *X. asphodeloides*. Our additional observations of germination after prolonged stratification show that embryo growth may occur slowly in the cold followed by dormancy release from stratification. Further testing is required to determine times and temperatures needed at each phase of dormancy treatment to break dormancy. Germination occurred in vivo for *X. tenax* when cold stratification for 14–16 weeks was applied with or without smoke-water treatment [40,41]. Fluridone, an ABA synthesis inhibitor, increased and speeded lily seed germination [42]. However, stratification (14 wks), smoke-water and fluridone did not assist *X. asphodeloides* germination in vitro. Our early cold temperature treatments likely did not break dormancy because the seed embryos were not sufficiently developed. The small undeveloped embryo present in fresh seed can be seen in Figure 2A.

The findings in this study provide a starting point for long term seed storage and production of *X. asphodeloides* plants for conservation and safeguarding (Table 11). Further studies are needed to improve, optimize and simplify procedures to germinate *X. asphodeloides* seeds and to grow planting stock. Major needs include: (1) Optimization of temperatures and discovery of minimum times required at each phase of dormancy treatment for maximum germination, (2) Simplification and optimization of methods to maintain planting mix moisture for optimal early plant growth.

With continued habitat destruction, over-collection, disease, and climate change, loss of *X. asphodeloides* populations are expected to continue. Developing methods for long-term seed storage and biodiversity maintenance are therefore critical. Procedures developed here provide tools to preserve germplasm and genetic diversity in *X. asphodeloides* and should be implemented immediately to assist in conservation of these beautiful plants.

## 4. Materials and Methods

### 4.1. Plant Materials, Experimental Design, and Evaluation

*X. asphodeloides* seeds were obtained from 17 sources (Table 12). After receipt, seeds were stored at room temperature in paper bags until use. Treatments were arranged in a completely randomized design. Data were analyzed by t-test or analysis of variance, and significant differences between treatments were determined by the Duncan’s multiple range tests using Statgraphics Plus V4.0 (Manugistics, Rockville, MD, USA).

### 4.2. X. asphodeloides Seed Sterilization and In Vitro Germination Experiments

Due to rarity of seeds, unless otherwise indicated, experiments typically consisted of 10–15 seeds from sources 1 or 2 for each treatment tested. Several factors to break seed dormancy were applied in tests for in vitro germination of seeds of *X. asphodeloides* [29]. Seeds were surface-sterilized for 10 min in 20% H_2_O_2_ as described by Ma et al. [23]. Scarified seeds were not surface-sterilized. After sterilization or scarification, seeds were placed on ½ strength MS salts (Murashige and Skoog 1962) with 3% sucrose, 100 mg/L myo-inositol, 0.50 mg/L thiamine HCl, 0.25 mg/L pyridoxine HCl, 0.25 mg/L nicotinic acid, and 1.0 mg/L glycine with or without an added plant growth regulator (PGR, see individual trials below). The pH of media was adjusted to 5.7 and solidified with 4.5 g/L Phytagel. Media were autoclaved at 121 °C for 20 min. Seeds were placed on 7 mL of medium in 60 × 15 mm Petri dishes. They were placed in a culture room at 24–25 °C under a 16/8-h (day/night) photoperiod with light supplied by cool white fluorescent lamps at an intensity of approximately 30 μmol m^−2^ s^−1^. Germination responses were examined weekly for two months. The following germination trials were carried out.

#### 4.2.1. Comparison of 1/3 vs. 1/2 MS Media Without PGRs

Seeds from source 1.

#### 4.2.2. Seed Scarification

In a first experiment, seeds not surface-sterilized were scarified in concentrated sulfuric acid for 1, 4, or 10 min followed by pouring seeds and acid into 200 mL of sterile distilled water for 3 min followed by five sterile water rinses for 5 min each. Source 1 seeds were placed on ½ MS medium without PGRs. In a second experiment Source 1 seeds were scarified for 3, 3.5, and 4 min in H_2_SO_4_. Source 2 seeds were scarified for 2, 3, 4, and 5 min, rinsed and placed on ½ MS medium without PGRs. Thirty seeds of source 2 were scarified for 4 min, rinsed and planted in greenhouse soil mix (1 part pumice: 3 parts perlite: 1 part milled sphagnum moss). Any germinants were transferred to fresh ½ MS medium without PGRs every 3–4 weeks.

#### 4.2.3. Seed Soaking in Gibberellic Acid (GA)

Seeds were surface sterilized and soaked overnight in 5-drop aliquots of filter-sterilized water solutions of 500, 750, or 1000 mg/L GA. Thirty seeds from source 1 placed on ½ MS medium without PGRs.

#### 4.2.4. Hot Water Treatments

Three hot water treatments varying in severity were tested to induce germination of source 1 seeds: (1) Ten seeds in a stainless-steel strainer were dipped for one second into boiling water. (2) Boiling water (100 mL) was poured over a strainer of 10 seeds for about 5 s. (3) Boiling water (100 mL) was removed from the heat and a strainer with 10 seeds was immersed into the water and allowed to cool to room temperature. Seeds were surface sterilized and placed onto ½ MS medium without PGRs.

#### 4.2.5. Seed Scarification Combined with Smoke Water

Source 2 seeds were scarified for 4 min and rinsed. Seeds were further soaked for 24 h in smoke water prepared from a smoke seed primer disc (FineBushPeople, Cape Town, South Africa) according to directions and placed on ½ MS medium without PGRs.

#### 4.2.6. 2 × 2 Factorial: Surface Sterilization or Scarification x ± Fluridone Soak Treatment

Surface-sterilized or scarified for 4 min. Half of the surface-sterilized or scarified seeds were not treated with fluridone. The other half were further soaked for 24 h in a filter-sterilized water solution of 2.4 µM fluridone. Ten to 25 resulting seeds of source 2 for each treatment were then placed onto ½ MS medium without PGRs.

#### 4.2.7. Seed Scarification and Germination in ½ MS vs. Full-Strength MS Salts

Twenty seeds from source 2 were scarified for 4 min. Half of the 20 seeds were placed onto ½ MS medium and half on full-strength MS medium for germination, each medium without PGRs.

#### 4.2.8. Seed Fluridone Treatment ± GA

Seeds from source 2 (15 per treatment) were placed on ½ MS medium without PGRs, ½ MS medium with 10 µM fluridone, or ½ MS medium with 10 µM fluridone and 100 µM GA.

#### 4.2.9. Seed Cold Treatment ± Scarification

Two sets of 60 seeds from source 2 were prepared for cold treatment. One set was surface sterilized and the other set was scarified for 4 min. All seeds were placed on ½ MS medium without PGRs and incubated in the dark at 4 °C. Ten plates, containing one seed each, were removed from the cold at 4, 6, 8, 10, 12, and 14 weeks for each set and incubated in the light.

#### 4.2.10. Seed Cold Treatment and Homemade Smoke Water with Surface Sterilization or 4 min Scarification

To prepare smoke water, plant debris collected from the base of wild *X. asphodeloides* plants was burned in a barbecue next to a bowl of distilled water. Thirty source 2 seeds were surface sterilized and 30 seeds were scarified for 4 min. All seeds were soaked in filter-sterilized smoke water for 24 h, placed on ½ MS medium without PGRs and incubated at 4 °C for 10, 12, or 14 weeks. Ten seeds from each treatment were removed at each cold treatment time and incubated in the light.

### 4.3. Tetrazolium Chloride Seed Viability Staining

We became concerned that seeds may not be viable. Tetrazolium chloride is commonly used to assay seed viability [43]. Source 1 and 2 seeds were soaked overnight in 1% tetrazolium chloride in distilled water. Seeds were sliced open and examined for red coloration indicating viability. Seeds scarified for 10 min were also tested for viability.

### 4.4. X. asphodeloides Embryo Isolation and In Vitro Germination Experiments

Seeds were soaked in distilled water for 48 h to soften the seeds and then surface sterilized. Embryos were dissected out of sterilized seeds in sterile conditions under a dissecting microscope. The seed was held base-up with forceps, while a scalpel was used to make a horizontal cut 2/3 across the seed without cutting either of the ends. The halves were then pulled apart to reveal a small oval shaped embryo. The embryo was carefully removed with forceps.

#### 4.4.1. Embryo Growth on ½ vs. Full Strength MS Medium ± 0.1 mg/L NAA

Isolated embryos were placed onto four test media: (i) ½ MS medium, (ii) ½ MS medium + 0.1 mg/L 1-Napthalenacetic acid (NAA), (iii) full strength MS medium without PGRs, (iv) full-strength MS medium + 0.1 mg/L NAA. Approximately 25 embryos from seed source 2 were isolated per treatment.

#### 4.4.2. Embryo Growth on ½ MS Medium with Different Sucrose Concentrations

Embryos were isolated from surface sterilized seeds from source 2 and placed onto media with different sugar concentrations. Approximately 18 isolated embryos per treatment were placed on ½ MS medium without PGRs varying in sucrose concentrations: 3%, 4%, 5%, 6%, 8%, and 10%.

Additional tests were run isolating embryos from seeds sources 1 (12 embryos per treatment) and 3 (20 embryos per treatment) with placement on ½ MS medium without PGRs containing 3 or 6% sucrose.

A larger trial was run comparing 3 and 6% sucrose using seed collected from five sites in Alabama (sources 2017-2, 6, 8, 9, and 10). Tests compared four or five replications of five isolated embryos per treatment and seed source.

### 4.5. X. asphodeloides Shoot Multiplication Experiments

Germinated seeds from trial 2 were transferred to ½ MS medium without PGRs monthly. The bulbous tissue and strap-like leaves that formed during germination continued to grow. After three months these masses were cut into two to four pieces and again placed on ½ MS medium without PGRs.

In the first shoot multiplication experiment, shoot cultures from the four source 1 germinants were divided into quarters (shoot clumps) and randomly placed onto four different media: (i) ½ MS without PGRs, (ii) ½ MS + 1 mg/L Kinetin, (iii) ½ MS + 2 mg/L trans-zeatin, (iv) ½ MS + 3 mg/L 6-benylaminopurine (BAP). Single shoot clumps were placed on 20 mL media in Magenta boxes (Magenta, Chicago, IL). Each test medium had nine replicates. All treatments were placed in the same lighted culture room used for germination. The number of shoots after five weeks was evaluated. 

In the second experiment shoots produced from the isolated embryos derived from sources 1, 2 and 3 were grown on ½ MS medium without PGRs. Ten shoots approximately 2 cm in size from each clone were placed onto each of six media containing 0 (control 1), 0.1, 0.2, 0.5 or 1.0 mg/L thidiazuron. To account for the possible effect of DMSO used in dissolving thidiazuron, a second control was added with 0.1 mg/L DMSO (control 2, the maximum amount of DMSO contained in the 1 mg/L thidiazuron treatment). All shoots were incubated under fluorescent light (cool white, light intensity at 30 µmoles/m^2^/s) at 24–25 °C and evaluated after four months without subculture.

An additional experiment (third experiment) was developed to optimize thidiazuron concentration. Ten shoots from each of four clones from seed sources 1, 2, and 3, each grown on ½ MS medium without PGRs, were each placed onto ½ MS medium containing 0, 0.05, 0.1, 0.15, or 0.2 mg/L thidiazuron. Shoots were grown for as described above and then evaluated after two months for the number of shoots smaller or larger than 3 cm.

Shoot cultures were maintained with 2–4 month subcultures over time on PGR-free medium or medium containing 0.15 mg/L thidiazuron.

### 4.6. X. asphodeloides Root Induction and Acclimation to Soil

Large shoots about 3 cm in length in PGR-free multiplication medium often formed roots in vitro spontaneously. In a preliminary test several spontaneously rooted shoots were rinsed to remove excess medium from the roots and planted into Atlanta Botanical Garden (ABG) new cutting mix (1 part milled New Zealand sphagnum moss: 1 part perlite: 1 part pumice).

Two trials were carried out to establish shoots in greenhouse potting mix. In the first trial shoots from two clones from seed sources 2 and 3 (DF-1 and LC-55) grown on medium with ½ MS medium without PGRs were separated into 10 (DF-1) or 12 (LC-55) shoots with roots and 10 shoots (DF-1 and LC-55) without roots. All shoots were dipped into Schultz Take Root rooting powder containing 0.1% indole-3-butyric acid (IBA) and planted about 6 mm deep into trays containing approximately 6 mm of horticultural grade charcoal and filled with a modified ABG new cutting mix (1 part milled sphagnum moss: 3 parts perlite: 4 parts pumice). Trays with plants were placed under transparent plastic domes, transferred to a greenhouse and watered daily. The survival rates were recorded after one and two months, and a few shoots were removed from containers to observe root development.

A second rooting trial was carried out. Shoots grown on ½ MS medium without PGRs were dipped into rooting powder with 0.1% IBA and planted into trays with horticultural grade charcoal and ABG new cutting mix. Four clones (TNF from source 1, DAW from source 2, and LC-51 and LC-57 from source 3) with 40 shoots each were tested.

#### In Vitro Root Induction

Another experiment was developed with four clones containing 20 shoots each. Shoots were split among four in vitro rooting treatments: (i) ½ MS without PGRs, (ii) ½ MS with 0.5 mg/L NAA, (iii) ½ MS with 1.0 mg/L NAA, (iv) ½ MS with 1.0 mg/L NAA plus 100 mg/L activated carbon (Sigma-Aldrich C-4386). Rooting percentages were evaluated at three, six, and eight weeks, and surviving plants with roots were evaluated at nine weeks.

Single shoots, 2–3 cm in size, were inserted about 5 mm into 20 mL test medium in Magenta boxes and incubated under fluorescent light (cool white, light intensity at 30 µmoles/m^2^/s) at room temperature (24–25 °C). In vitro rooting was evaluated after 1–2 months. Nine weeks after placement in medium, shoots were removed from the medium, rinsed with water, and planted with or without roots into 5 cm x 5 cm pots with approximately 1 cm charcoal and potting mix containing 3 parts modified cutting mix (1 part sphagnum moss: 1 part pumice: 4 parts perlite) plus 1 part modified ABG Carnivorous plant mix (5 parts peat moss: 1 part sphagnum moss: 1 part builder’s sand). Potting mix was tightly packed and shoots were inserted about 1 cm into the mix. Trays with plants were placed under transparent plastic domes and placed in a greenhouse in early summer, watered daily, and observed weekly.

To evaluate different types of soil substrate we planted five rooted shoots in a local mix from the State Botanical Garden of Georgia (SBG1) that contained pine bark. SBG1 potting mix contained 1 part orchid bark mix, 1 part builder’s sand, and 1 part SBG2 pine bark mix. SBG2 pine bark mix contained 125 gallons fine grade pine bark, 8 cubic ft coarse vermiculite, 8 cups dolomitic limestone, 2 cups superphosphate, 1 cup each of gypsum, potassium nitrate, calcium nitrate, and Micromax granular micronutrient fertilizer. Individual plants were placed in 4 cm pots with a bottom layer of horticulture grade charcoal. Medium was washed from the roots and plants were carefully planted in the soil and covered with a humidity dome for two weeks. To keep soil drier instead of drenching pots daily, individual pots were watered with 15–20 mL water every other day and provided with an extra 10 mL prior to weekends. After about four weeks water was further reduced to 10 mL every other day.

To determine if beneficial organisms from native *X. asphodeloides* soil help rooted shoots survive, we compared rooted shoot survival in two soil mixes without or with 10% amendment of native soil collected from the base of wild *X. asphodeloides* plants. Soil cores were collected to a depth of 12 inches adjacent to healthy plants from four AL sites. Large rocks, roots, and debris were removed and equal amounts of soil were mixed together from each site. ABG modified new cutting mix (1 part sphagnum moss, 3 parts perlite, 4 parts pumice) or SBG 1 mix were left as is or amended with 10% native *X. asphodeloides* soil forming four treatments. For each treatment five rooted shoots of clone TNF-7 and five shoots of clone TNF-115 were each planted in 4 cm pots with a bottom layer of horticultural grade charcoal and filled with the soil mix to be tested. 

### 4.7. X. asphodeloides Acclimated Greenhouse Plants Planted in the Field

Thirty plants from rooted shoots grown in the greenhouse for six months or longer, resulting from the trials to evaluate soil types above, were planted at field site 11 in the Talladega National Forest. A hilltop near site 11 and resembling natural *X. asphodeloides* habitat was located and divided into four quadrants. Six to nine plants were planted into each quadrant in February, 2019.

### 4.8. X. asphodeloides Seed Cryopreservation

Twenty seeds from source 2 were used for the first cryopreservation experiment. Seeds were placed 10 seeds per vial into 2 mL Nalgene cryogenic storage vials (Thermo Scientific, Waltham, MA, USA) and rapidly immersed in liquid nitrogen (LN). After 48 h the vials were removed from LN and re-warmed in a 37 °C water bath for 1–2 min (Lynch et al. 2013 [24]). Whole seeds were surface sterilized and embryos were isolated and placed on ½ MS medium without PGRs.

Five seeds from source 3 after 13 months of storage at room temperature were tested for water content. Seeds were placed in small pre-weighed glass vials, covered with aluminum foil to prevent water exchange with the air, weighed, dried for 24 h at 105 °C, weighed again, and water contents were calculated.

Fifteen seeds from source 3 were placed into each of two cryovials. One vial was rapidly immersed into LN and the other was held at room temperature. After 48 h, the vial was removed from LN and re-warmed in a 37 °C warm water. Both sets of 15 seeds were soaked for 24 h in sterile water at 4 °C. Seeds were surface sterilized and embryos were isolated and placed on ½ MS medium without PGRs.

Another cryopreservation experiment was carried out with seed sources 4 and 5, they were stored at room temperature for 3–5 months in paper bags until tested for water content and cryopreservation using isolated embryos. Water content measurements were determined for five seeds from each source. About 350 seeds were used, inserting 30 seeds per cryovial. They were rapidly immersed in LN. A similar number of seeds remained at room temperature for the control.

Additional seeds from sources 2, 3, 4, and 5 were evaluated to determine survival after varying periods of time at room temperature or storage at room temperature followed by cryopreservation (Table 8). Experiments evaluated 32–33 seeds per treatment and storage time using the embryo isolation method.

### 4.9. X. asphodeloides Germination of Whole Seeds with Treatment for Double Dormancy

To simplify germination from cryopreserved or non-cryopreserved seeds and to produce tissue more easily for micropropagation, we returned to whole seed germination. Prior greenhouse trials for *X. asphodeloides* showed that slow seed germination occurred over two years [19,22,39]. We hypothesized that *X. asphodeloides* seeds have double dormancy and require a warm moist exposure to cause embryo development followed by a cold period to break dormancy followed by a moist, warm environment for root and shoot growth. To test this hypothesis 30 non-cryopreserved seeds from sources 2017-3 and 2017-5 were each tested in two treatments: (1) whole sterilized seeds were placed in individual Petri dishes with ½ MS medium containing 3% sucrose without PGRs; and (2) whole sterilized seeds were placed into individual 14 × 14 cm trays containing soil mix composed of one part perlite and one part aged Monterey pine bark. All seeds were incubated at room temperature in darkness for 12 weeks followed by 12 weeks of incubation at about 4–5 °C. After this period, they were transferred to the lighted culture room as described above for eight weeks. Soil mix trays were misted every few days to maintain soil moisture. Petri plates were inspected weekly and seeds showing visible contamination were recorded and discarded.

Knowing that dry seeds lose viability over several years, 10 leftover seeds from a mix of source 2017 sites were stored in moist sphagnum moss at 4–5 °C for 3 ½ months.

To evaluate the viability after long term storage at room temperature, seeds from sources 2017-1–11 stored dry at room temperature for 8 months were placed in wet sphagnum moss in Ziplock bags and kept dark at 4–5 °C for 16 weeks. Germinants were planted into SBG1 soil in 10 cm pots with up to ten per pot and placed on grow shelves lighted with cool white fluorescent light and under humidity domes for the next 2–3 weeks. Humidity domes were removed and pots were watered daily with about 3 mL per pot for the next two weeks. Pots were then watered every other day with about 4 mL per pot to allow the soil surface to dry. This watering regime was continued for the next six months until plants were transplanted into individual containers.

## Figures and Tables

**Table 1:** *Xerophyllum asphodeloides* embryo germination percentage for three seed sources (1, 2, and 3) on ½ MS medium without PGRs with different sucrose concentrations after eight weeks.

Seed Source and Medium Sucrose Content	Number Embryos Tested	Embryo Germination (%)
Source 2		
3% sucrose	18	50 a
4% sucrose	18	61 a
5% sucrose	18	61 a
6% sucrose	18	67 a
8% sucrose	18	50 a
10% sucrose	18	33 a
Source 1		
3% sucrose	12	42 a
6% sucrose	12	33 a
Source 3		
3% sucrose	20	35 a
6% sucrose	20	55 a

Values followed by the same letter for a seed source are not statistically different by the multiple range test at *p* < 0.05.

**Table 2:** *Xerophyllum asphodeloides* embryo germination percentages after seven weeks for different source sites (2017) on ½ MS medium with different sucrose concentrations.

Seed Source Site (2017)	Number of Non-Contaminated Explants for Media Containing 3 and 6% Sucrose ^2^	Embryo Germination (%)	
		3% Sucrose	6% Sucrose
2	17, 16	87.5 ± 7.2	65.0 ± 11.9
6	25, 25	56.0 ± 7.5	44.0 ± 11.7
8	20, 20	61.3 ± 9.7	72.5 ± 11.1
9	23, 21	73.3 ± 4.2	58.0 ± 6.6
10	25, 21	64.0 ± 11.7	74.0 ± 9.7
Average ^1^		68.6 a	62.7 a

^1^ Values followed by the same letter are not statistically different by the multiple range test at *p* = 0.05. Analyses are based on arcsine √% transformation. ^2^ Non-contaminated explants did not show visible contamination.

**Table 3:** *Xerophyllum asphodeloides* shoot multiplication over four months for three seed sources (1, 2, and 3) on ½ MS medium without PGRs varying in thidiazuron content.

Thidiazuron and DMSO Content	Number of New Shoots Per Explant
No thidiazuron, no DMSO (control 1)	4.3 a
No thidiazuron, 0.1 mg/L DMSO (control 2)	6.6 ab
Thidiazuron 0.1 mg/L, DMSO 0.1 mg/L	15.0 b
Thidiazuron 0.2 mg/L, DMSO 0.1 mg/L	9.8 ab
Thidiazuron 0.5 mg/L, DMSO 0.1 mg/L	5.6 ab
Thidiazuron 1.0 mg/L, DMSO 0.1 mg/L	8.3 ab

Values followed by the same letter are not statistically different by the multiple range test at *p* = 0.05.

**Table 4:** *Xerophyllum asphodeloides* shoot multiplication over two months for three seed sources on ½ MS medium varying in thidiazuron content.

Thidiazuron	Average # of Shoots Grown Per Starting Shoot		
	<3 cm	>3 cm	Total
None	0.5 a	1.0 a	1.5 a
0.05 mg/L	1.7 b	0.9 a	2.7 bc
0.1 mg/L	1.4 b	0.7 a	2.3 ab
0.15 mg/L	1.8 b	1.4 b	3.3 c
0.2 mg/L	1.9 b	1.1 ab	2.9 bc

Values within the same column followed by the same letter are not statistically different by the multiple range test at *p* = 0.05.

**Table 5:** *Xerophyllum asphodeloides* shoot performance during rooting and acclimation to soil.

Seed Source and Treatment	Percentage Surviving		
	1 Month	2 Months	4 Months
Source 2 (DF-1) non-rooted	80%	60%	30%
Source 2 (DF-1) rooted	90%	90%	40%
Source 3 (LC-55) non-rooted	100%	100%	20%
Source 3 (LC-55) rooted	100%	92%	67%

**Table 6:** *Xerophyllum asphodeloides* shoot rooting percentage after placement in in vitro rooting medium containing ½ MS and varying amounts of NAA and activated carbon.

Treatment and Clone	3 Wks Rooting	6 Wks Rooting	8 Wks Rooting	9 Wks Rooted and Surviving
Control, no PGRs—DAW	0%	0%	20%	0%
Control, no PGRs—LC51	0%	0%	0%	0%
Control, no PGRs—LC57	20%	0%	20%	0%
Control, no PGRs—TNF	40%	60%	80%	40%
Average	15%	15%	30% a	10%
0.5 NAA—DAW	0%	80%	100%	20%
0.5 NAA—LC51	60%	80%	80%	80%
0.5 NAA—LC57	20%	80%	100%	80%
0.5 NAA—TNF	20%	60%	80%	80%
Average	25%	75%	90% c	65%
1.0 NAA—DAW	40%	40%	40%	0%
1.0 NAA—LC51	40%	40%	40%	40%
1.0 NAA—LC57	20%	100%	100%	0%
1.0 NAA—TNF	80%	40%	40%	20%
Average	45%	55%	55% b	15%
1.0 NAA + AC—DAW	40%	20%	0%	0%
1.0 NAA + AC—LC51	20%	0%	0%	0%
1.0 NAA + AC—LC57	20%	0%	20%	40%
1.0 NAA + AC—TNF	20%	40%	20%	0%
Average	25%	15%	10% a	10%

Average values within the 8 week data column are statistically different by the multiple-range test at *p* < 0.01 as indicated by different letters.

**Table 7:** *Xerophyllum asphodeloides* isolated embryo germination after 8 weeks for control and seed cryopreserved for one month.

Seed Source and Treatment	# of Starting Seeds	# Contaminated Seeds	Isolated Embryo Germination (%)
Seed source 4			
Control	25	0	56% a
Cryopreserved	25	0	48% a
Seed source 5			
Control	23	5	61% a
Cryopreserved	42	5	62% a

Values within a trial followed by the same letter are not statistically different by the multiple range test at *p* = 0.05.

**Table 8:** *Xerophyllum asphodeloides* isolated embryo germination in vitro after long term seed storage at room temperature or storage at room temperature followed by cryopreservation.

Seed Source and Treatment	Number Of Starting Seeds	Number of Contaminated Seeds	Number/total (%) of Isolated Embryos Germinating after 3 Months on Medium
Seed source 2 (DF, collected October 2010)			
3 years at RT	33	0	0%
Seed source 3 (LC, collected November 2010)			
3 years at RT	32	0	0%
Seed source 4 (TNF, collected August 2012)			
15 months at RT	10	1	22%
5 mo at RT + 20 months CP	32	24	63%
Seed source 5 (DF, collected October 2012)			
12 months at RT	10	1	11%
24 months at RT	33	0	6%
4 mo at RT + 20 months CP	33	0	67%

mo = months, RT = room temperature, CP = cryopreserved.

**Table 9:** *Xerophyllum asphodeloides* whole seed germination on medium in vitro or in greenhouse soil after treatment to remove double dormancy ^1^.

Seed Source	Contamination In Vitro # of Seeds (%)	Non-Contaminated Seed Germination on Medium # of Seeds (%)	Overall Germination In Vitro *#* of Seeds (%)	Germination in Greenhouse Mix # of Seeds (%) ^2^
2017-3	16/30 (53%)	14/14 (100%)	14/30 (47%) a	15/30 (50%) a
2017-5	12/30 (40%)	16/18 (89%)	16/30 (53%) a	18/30 (60%) a

^1^ Double dormancy treatment—12 weeks in moist medium or soil in dark at room temperature followed by 12 weeks of 4–5 °C in dark followed by incubation in a lighted culture room at room temperature. ^2^ Values followed by the same letter in a row are not significantly different using a *t*-test.

**Table 10:** *Xerophyllum asphodeloides* seed germination after 14–16 weeks of stratification at 4–5 °C in wet sphagnum moss.

Seed Source	Number of Seeds Stratified	Number of Seeds Germinating (%)
2017-1	44	23%
2017-2	44	9%
2017-3	44	0%
2017-4	35	11%
2017-5	44	18%
2017-6	44	0
2017-7	44	14%
2017-8	88	23%
2017-9	44	11%
2017-10	50	2%
2017-11	5	20%

**Table 11:** Recommended steps for *Xerophyllum asphodeloides* seed cryopreservation, germination of isolated embryos or seeds and growing plants.

Seed cryopreservation
(1) Determine seed moisture content and if seeds need to be dried (2) Place seeds in cryogenic storage vials (3) Rapidly immerse vials in liquid nitrogen (LN) (4) Remove vials from LN and re-warm in a 37 °C water bath for 1–2 min (5) Germinate seeds in vitro or in vivo
Seed germination in vitro (production of initial shoots for micropropagation)
Option A (whole seed germination over 26–28 weeks, low labor requirement)
(1) Surface sterilize fresh of cryopreserved seeds (2) Place on ½ MS medium without PGRs in sterile containers (3) Incubate in dark for 12 weeks at room temperature (4) Incubate in cold (4 °C) in dark for 16 weeks (5) Move containers to lighted culture room for germination (6) Remove germinants and use for micropropagation
Option B (embryo isolation, germination in about 2–4 weeks, high labor and skill required)
(1) Surface sterilize fresh of cryopreserved seeds (2) Remove embryo under sterile conditions (3) Place embryo on ½ MS medium without PGRs and use for micropropagation
Seed germination in vivo
(1) Place fresh or cryopreserved seeds in plastic bag with moist sphagnum moss at room temperature for 12 weeks (2) Move bags to 4 °C in dark for stratification for 16 weeks (3) Move bags to light at room temperature. As germinants occur transplant to SBG1 (4) Cover with humidity domes for 2–4 weeks. Water sparsely with about 3 mL per 10 cm pot daily for 2 weeks. Water with about 4 mL every other day allowing soil surface to dry. Continue for about 6 months and transplant individual plants into larger pots.
Micropropagation
(1) Place contamination-free germinant or shoot on ½ MS medium without PGRs (2) Subculture and divide shoots as needed until desired number of shoots are obtained (3) Root shoots in PGR free medium, ½ MS with 0.5 mg/L NAA or dip shoots in rooting powder (4) Rinse medium from rooted shoots. Insert rooted shoots or rooting powder treated shoots carefully into SBG1. Cover with humidity domes for 2–4 weeks. Water sparsely with about 3 mL per 10 cm pot daily for about 4 weeks. Water with about 4 mL every other day allowing soil surface to dry. Continue for about 6 months and transplant individual plants into larger pots.

**Table 12:** Seed sources and sampling times for *X. asphodeloides*.

Seed Source	Collection Location	Harvesting Time	Seed Condition
1	Talladega National Forest (TNF), AL	6 August 2010	Seeds showed visible white fungal hyphae on coat surface
2	Dawson Forest (DF) in Dawson County, GA	October 2010	--
3	Canton, Cherokee County (LC), GA	November 2010	--
4	TNF	27 August 2012	--
5	DF	22 October 2012	--
6	TNF	12 December 2013	--
2017-1 to 11	TNF Shoal Creek Division	Fall 2017	--

## Data Availability

Not applicable.
